# Factors associated with inflamm-aging in institutionalized older people

**DOI:** 10.1038/s41598-021-97225-3

**Published:** 2021-09-15

**Authors:** Leônidas de Oliveira Neto, Vagner Deuel de O. Tavares, Pedro Moraes Dutra Agrícola, Larissa Praça de Oliveira, Márcia Cristina Sales, Karine Cavalcanti Maurício de Sena-Evangelista, Igor Conterato Gomes, Nicole Leite Galvão-Coelho, Lúcia Fátima Campos Pedrosa, Kenio Costa Lima

**Affiliations:** 1 Department of Arts, Postgraduate Program in Rehabilitation Sciences, Federal University of Rio Grande do Norte, Av. Senador Salgado Filho, 3000-Lagoa Nova, Natal, RN 59078-970 Brazil; 2 Department of Physiology and Behavior, Federal University of Rio Grande do Norte, Natal, RN Brazil; 3 Department of Physical Education, Maurício de Nassau College, Natal, RN Brazil; 4 Graduate Program in Collective Health, Federal University of Rio Grande do Norte, Natal, RN Brazil; 5 Graduate Program in Health Sciences, Federal University of Rio Grande do Norte, Natal, RN Brazil; 6 Department of Nutrition, Federal University of Rio Grande do Norte, Natal, RN Brazil; 7 Department of Epidemiology, School of Public Health, University of São Paulo, Brazil, São Paulo, SP Brazil

**Keywords:** Physiology, Biomarkers, Health care, Risk factors

## Abstract

The increase in inflammatory cytokines associated with a reduction in the bioavailability of zinc has been used as a marker for inflammation. Despite the high inflammatory state found in institutionalized older individuals, few studies have proposed verifying the factors associated with this condition in this population. To verify the factors associated with inflamm-aging in institutionalized older people. A total of 178 older people (≥ 60 years old) living in nursing homes in Natal/RN were included in the study. Cluster analysis was used to identify three groups according to their inflammatory state. Analysis anthropometric, biochemical, sociodemographic, and health-related variables was carried out. In sequence, an ordinal logistic regression was performed for a confidence level of 95% in those variables with p < 0.20 in the bivariate analysis. IL-6, TNF-α, zinc, low-density lipids (LDL), high-density lipids (HDL), and triglycerides were associated with inflamm-aging. The increase of 1 unit of measurement of LDL, HDL, and triglycerides increased the chance of inflammation-aging by 1.5%, 4.1%, and 0.9%, respectively, while the oldest old (≥ 80 years old) had an 84.9% chance of presenting inflamm-aging in relation to non-long-lived older people (< 80 years). The association between biochemical markers and inflamm-aging demonstrates a relationship between endothelial injury and the inflammatory state. In addition, the presence of a greater amount of fat in the blood may present a higher relative risk of death.

## Introduction

The aging process occurs parallel to the progressive deterioration in cells and functioning of the organs due to metabolic, immunological, neuroendocrine, or oxidative stress^1,2^. These alterations during aging are explained by the imbalance between oxidant/antioxidant^2^ and inflammatory/anti-inflammatory mechanisms resulting from the pro-inflammatory state known as *inflamm-aging*^3^.

*Inflamm-aging* is a process that occurs concomitantly with aging and is related to the presence of a mild and chronic inflammatory state. A moderate inflammatory response is beneficial to the body, but when excessive, the response becomes harmful. For example, alterations are triggered by exacerbated production of proinflammatory cytokines such as interleukin-6 (IL-6), tumor necrosis factor alpha (TNF-α), and interleukin 1β (IL-1β)^4,5^.

In addition, the decline in zinc bioavailability is a process that occurs in aging parallel with exacerbation of the inflammatory state^6,7^. Zinc (Zn) deficiency in older people could result from a decrease in Zn intake, medication use, malabsorption syndromes, or kidney diseases^8^. It is known that zinc has important roles in several cellular functions, such as in the oxidative stress cellular system, where it protects the cells from damage by free radicals. This element is also important for regulation of many physiological systems and processes, for instance blood clotting, through increases in the aggregation of circulating platelets, in synthesis, secretion, and insulin signaling, and also in aiding bone homeostasis and thyroid and immune function^8–12^.

There is a growing body of literature suggesting that zinc plays a relevant role in some pathways of the acquired immune system by activating the anti-inflammatory Signal Transducers and Activators of Transcription 3 (STAT3) or by inhibiting the proinflammatory Nuclear Factor kappa-light-chain-enhancer of activated B cells (NF-κB) pathway. Therefore, the equilibrium of Th1/Th2 shifts the immune response towards the anti-inflammatory Th2 system^9,13,14^.

At physiological levels, zinc acts mainly by inhibiting the proinflammatory NF-κB pathway and, consequently, proinflammatory cytokines (IL-1, IL-6, and TNF-α), through several mechanisms and at many levels, for instance, upregulating A20 gene expression, increasing peroxisome proliferator-activated receptor alpha (PPAR-α), or inhibiting nucleotide phosphodiesterase (PDE)^14,15^. Moreover, as zinc deficiency is usually observed in aging, there is a hypothesis suggesting a close relationship between *inflamm-aging* and zinc deficiency^13^.

A robust body of evidence has shown that inflammation is present independent of whether older people are affected by diseases or are healthy^16^. It seems that inflammation is characteristic of both successful and unsuccessful aging. However, inflammation has been linked to various diseases, such as Alzheimer's disease^17^, Parkinson’s disease^18^, cardiac disease^19^, osteoporosis and insulin resistance^20^, chronic respiratory disease^21^, and cancer^22^, where in some cases zinc deficiency has also been associated^23^.

In addition, it seems that inflammation is related to high mortality rates^24^, especially in institutionalized older people, who present even higher rates^25^. This is possible because institutionalized older people have worse health status indicators, with a high prevalence of high inflammatory stress. These patients showed comorbidities such as hypertension^26^, diabetes^27^, and kidney disease^28^, and very high levels of systemic inflammatory biomarkers^29,30^.

Although a relationship has been established between inflammatory cytokine elevation and zinc reduction, with the onset of *inflamm-aging*, studies that explore this condition through the association of these markers are still scarce, especially in institutionalized older people, probably due to the high price of biomarker tests. However, it is extremely important to assess the factors associated with inflammation so that we can more easily trace the risk profile of these institutionalized older people and thus think more easily about protection strategies for this population.

Thus, the objective of the present study was to assess the blood profile in institutionalized older people for characterizing *inflamm-aging*.

## Methods

### Study design and population

The study was conducted in 304 older people (≥ 60 years old) of both sexes, living in long-term care institutions (LSIE) for older people, for or not for profit, and registered in the Health Surveillance Agency (ANVISA), Brazil. The following individuals were excluded from the initial sample: older people who did not reside in LSIEs or those from whom it was difficult to obtain venous access, preventing blood collection to characterize the inflammatory state and biomarker variables (n = 95). The project was approved by the Ethics and Research Committee of the Federal University of Rio Grande do Norte (CEP/UFRN), protocol 263/11; CAAE 0290.0.051.000-11. All experiments were performed in accordance with relevant guidelines and regulations. After explaining the methodological procedures and objectives of the study, all participants signed the Informed Consent Term prior to the beginning of the data collection.

### Biomarkers, anthropometric and sociodemographic variables, and health-related factors

#### Biomarker analysis

IL-6 and TNF-α were used to identify a possible inflammatory profile based on other studies about inflammation^31^. IL-6, TNF-α, and CRP (C-reactive protein) are the gold standards of systemic inflammatory biomarkers^32,33^. Since IL-6 is the main trigger to liver release of CRP, their synthesis and release are highly correlated^32^. Whereas IL-6 and TNF-α are specific pro-inflammatory biomarkers, CRP is unspecific. Therefore, measuring CRP is preferred when there are financial or logistic restrictions to dosage of IL-6 and TNF-α. On the other hand, Zinc was chosen as an anti-inflammatory biomarker because there is a growing body of literature suggesting that zinc plays a relevant role in the shifting of the immune response towards the anti-inflammatory Th2 system^9,13,14^.

Blood was collected by venipuncture after a 12–14 h overnight fasting period. To minimize mineral contamination, all glassware and plastic containers used during the zinc blood collection and analysis were carefully demineralized in a 20% nitric acid bath for at least 12 h and rinsed ten times with ultra-pure water^34^ (*Direct*-*Q*^®^ 3 Water Purification Systems, Merck Millipore, Darmstadt, Germany). Blood samples for zinc analysis were placed in demineralized tubes containing 100 μL of 30% sodium citrate solution and separated into plasma. We calibrated the assay using the following working conditions: wavelength, 231.9 nm; slit width, 1.0 nm; current, 5.0 mA; expansion factor, 1.0; and sample flow, 5 mL/min. A standard zinc solution (Tritisol; Merck) was used to define the points of the calibration curve, which included concentrations of 0.10, 0.25, 0.50, 0.75, and 1.00 μg/mL^34^. The standard curve was prepared with the addition of 5% glycerol. Seronorm Trace Elements Serum L-1 solution (SERO AS, Billingstad, Norway; Reference 201405, Lot 0903106) was used as a reference for zinc analysis. Plasma zinc concentrations were determined according to the method described by Rodrigues et al.^35^. We used values of 70–110 µg Zn/dL as the reference for plasma zinc concentration^36^.

Blood samples for lipid profile analysis were placed in tubes without anticoagulant and analyses were performed by the colorimetric method^37^ (Labtest Diagnóstica^®^ kits). The levels of total cholesterol, HDL (g/dL), LDL (g/dL), triglycerides (mg/dL), albumin (g/dL), urea (mg/dL), and creatinine (mg/dL) were determined by means of colorimetric enzymatic assays using the Bio-200 Biochemical Analyzer (Barueri, Brazil) and Labtest Diagnostic^®^ kits.

Immunologic analyses were conducted via chemiluminescence using an Immulite 1000^®^ device (Malvern, PA, USA) with kits from Siemens^®^ (Siemens Healthcare Diagnostics Products, Llanberis, Caernarfon, UK). For CRP, the cut-off point of 10 mg/dL was adopted^34^. For tumor necrosis factor (TNF) alpha, the recommended reference range of serum TNF-α was from undetectable to 8.1 pg/mL^38^. For interleukin 6 (IL-6) the recommended reference range of IL-6 was to < 3.1 pg/mL^39^. In this case, only samples with an intra assay coefficient of variation (CV) of less than 5% and inter assay coefficient of variation (CV) of less than 10 were considered in the study. CRP, TNF-α, and IL-6 assays were validated for a dilution factor of 1:101, 1:128, and 1:50, respectively, in diluent reagent.

#### Anthropometric measures

From the anthropometric measures, the body mass index, calf circumference, arm muscle area (AMA), and arm fat area (AFA) were calculated. The ratio of body weight (kg) to height (m) squared was used to calculate BMI^40^. The perimeter of the arm and tricipital skinfold, both measured at the midpoint between the acromion and olecranon of the ulna, were used to calculate the AMA and AFA, using the equation proposed by Frisancho^41^.

#### Sociodemographic variables

Data on sex, age, type of institution, and number of drugs and morbidities were obtained from the records of the participants at each institution. The variable "type of institution" was selected as a *proxy* for the economic situation of the older people, considering the hypothesis that the current health status of the older people reflects their past social and economic conditions. These conditions may determine the choice of the type of institution, whether for profit or not.

### Statistical analysis

To group the subjects according to the inflammatory state, some criteria were defined to analyze the variables to be grouped, followed by the analysis of the absence of multicollinearity between the variables that served as the basis for the construction of the *clusters*, from the *Pearson* correlation coefficient. The standardization of the variables was performed, transforming the data into Z-scores to standardize and prevent distortion of the structure of the clusters. Subsequently, a linear regression was performed to identify and remove the possible *outliers* through the Mahalanobis distance.

After finalizing this step, cluster analysis was conducted with the objective of grouping the older people with similar inflammatory characteristics. For this, the clustering algorithm was used through the *k-means* method with the objective of finding a data partition with reduced Euclidean distance to the center of the cluster, aiming to form homogeneous groups from the predetermined characteristics of the zinc, IL-6, and TNF-α levels.

In this process, a pertinent number of three clusters was verified, considering that for these conglomerates, each cluster included participant numbers > 30. After the creation or identification of the clusters of the inflammatory state of the study subjects, the parametric status of the independent variables was confirmed by the *Kolmogorov–Smirnov* test*,* and the ANOVA test with Bonferroni post hoc or *Kruskal–Wallis*, followed by the Mann–Whitney test with penalization, carried out to compare the variables (biomarkers, anthropometric, sociodemographic, and health of the older people) between the clusters. The chi-squared test was used to verify the association between the qualitative variables, sex, type of LSIE, polypharmacy, multi-morbidities, and clusters. The multicollinearity of the variables that obtained a value of p < 0.20 in these analyses was tested, and then an ordinal logistic regression was performed for a 95% confidence level.

### Ethical approval

All experiments were performed in accordance with relevant guidelines and regulations.

## Results

Of the nine institutions participating in the study, five were not for-profit and four were for-profit. From the total of 304 older residents in the institutions, 24 older people or their guardians refused to participate in the study, and 71 participants were excluded because they met some of the exclusion criteria. Older people who presented extreme values (coefficient variation error) of biochemical variables (*outliers*) (n = 7) or did not perform all evaluations (n = 24) were excluded. Prior to the grouping of the older people according to characteristics of their inflammatory state, the data were analyzed to confirm the validity of the multivariate analysis, in which one individual was identified as an *outlier* (*Mahalanobis* Distance-D2) and eliminated from the sample, leaving a final sample of 178 older people.

Three clusters (n > 30) were formed, presented in Table 1, which demonstrates the mean values and differences in the inflammatory variables for each group formed. When analyzing the variables used as a basis for the composition of the clusters, Zn (F = 116.83) showed the best discrimination between clusters, followed by IL-6 (F = 101.09) and TNF-α (F = 72.98). With respect to Cluster 1, higher concentrations, outside the reference values were observed for IL-6 and TNF-α, associated with a low concentration of Zn, in relation to cluster 3. Cluster 2 showed an intermediary state compared to the other clusters, with concentrations of inflammatory biomarkers slightly above the reference limit and only TNF—α levels were higher than the no inflammatory state group (Cluster 3). The Zn levels in cluster 2 were like cluster 1, that is, inside the reference limits but higher than cluster 3. Cluster 3 presented an antagonistic characteristic to cluster 1, with a lower concentration of IL-6 and TNF-α and a higher concentration of Zn, meaning a low inflammatory load.

**Table 1:** Characterization of the inflammatory state distributed into clusters formed by inflammatory cytokines and zinc in the plasma of institutionalized older people.

Conglomerates	N	IL-6 (pg/mL)^d^	TNF-α (pg/mL)^d^	Zn (μg/dL)^e^	Cluster description
Cluster 1	40	**8.15 (6.8–9.77)**^**a**^	**12.6 (9.8–14.8)**^**a**^	76.27 (11.53)^a^	High inflammatory state
Cluster 2	70	**3.25 (2.2–4.8)**^**b**^	**8.2 (4.9–8.7)**^**b**^	74.08 (8.60)^a^	Mild inflammatory state
Cluster 3	77	**2.9 (2.2–3.9)**^**b**^	5.1 (4.0–8.1)^c^	98.11 (9.94)^b^	No inflammatory state
p-value		< 0.001	< 0.001	< 0.001	
Reference values		< 3.1 pg/mL	< 8.1 pg/mL	70–110 μg/dL	

*IL-6* interleukin 6, *TNFα* tumor necrosis factor α, *Zn* zinc.

Bold text indicates values outside the reference levels.

^a/b/c^Different letters indicate a statistically significant difference between the variables.

^d^Data are presented as median and interquartile range considering nonparametric data.

^e^Data are distributed as mean and standard deviation considering parametric data.

The association between the independent variables and inflammatory states of these clusters was measured, in which it was possible to observe a difference (p < 0.05) between the clusters for the variables total cholesterol, LDL, triglycerides, and creatinine (Table 2). The high inflammatory state, cluster 1, presented a higher level of total cholesterol, LDL, triglycerides, and creatinine in relation to cluster 3, no inflammatory state, demonstrating the same antagonistic relation presented between the inflammatory markers. The mild inflammatory state, cluster 2, presented characteristics similar to the high inflammatory state cluster in the variables total cholesterol, LDL, triglycerides, and creatinine. The latter variable, in turn, also presented a similar response to the no inflammatory state cluster, indicating a smaller effect in relation to the inflammatory variables present in each cluster.

**Table 2:** Associations between biochemical variables and inflammatory states in institutionalized older people.

Independent variables^d^	High inflammatory state (Cluster 1) Mean (SD)	Mild inflammatory state (Cluster 2) Mean (SD)	No inflammatory State (Cluster 3) Mean (SD)	*p-value*
Age (years)	80.4 (8.87)	81.07 (9.64)	83.02 (8.67)	0.816
Total Cholesterol (mg/dL)	186. 23 (40.79)^a^	185.70 (39.52)^a^	149.56 (34.23)^b^	< 0.001*
HDL (g/dL)	45.08 (7.63)	44.97 (10.19)	42.41 (9.93)	0.194
LDL (g/dL)	116.82 (37.56)^a^	113.84 (36.77)^a^	87.46 (31.55)^b^	< 0.001*
Triglycerides (mg/dL)	121.85 (51.46)^a^	134.64 (53.1)	98.60 (42.47)^b^	< 0.001*
Urea (mg/dL)	40.5 (13.3)	41.3(15.7)	38.8(15.6)	0.607
Albumin (g/dL)	3.58 (0.75)	3.64 (0.74)	3.46 (0.75)	0.334

Independent variables^e^	Cluster 1 Median (IQR)	Cluster 2 Median (IQR)	Cluster 3 Median (IQR)	*P-value*
Total proteins (mg/dL)	7.10 (6.4–7.8)	7.30 (6.9–7.7)	7.10 (6.6–7.7)	0.454
Creatinine (mg/dL)	1.0 (0.9–1.2)^a^	0.9 (0.8–1.1)^a/b^	0.9 (0.8–1.1)^b^	0.034*

*p < 0.05.

^a/b/c^Different letters denote a statistically significant difference between the variables.

^d^Data are presented as median and interquartile range considering nonparametric data.

^e^Data are distributed as mean and standard deviation considering parametric data.

In Table 3, it can be seen that older people living in for-profit LSIEs are predominantly in the mild inflammatory state (46.3%) and no inflammatory state (35.0%) clusters, whereas those residing in not for-profit LSIEs are predominantly in the cluster no inflammatory state (53.1%). Regarding the distribution by sex, male individuals are predominantly in the cluster with no inflammatory state (56.1%) in relation to the clusters with high inflammatory state (22%) and mild inflammatory state (22%), while women are predominantly found in the clusters mild inflammatory state (41.8%) and no inflammatory state (37%) in relation to the cluster with high inflammatory state (21.2%). In addition, there were no differences between clusters regarding BMI, age, number of morbidities, and polypharmacy.

**Table 3:** Association of inflammatory states with sex, age, BMI, morbidities, polypharmacy, and type of LSIE of institutionalized older people.

Variables*	High inflammatory state, (Cluster 1) N (%)	Mild inflammatory state, (Cluster 2) N (%)	No inflammatory state, (Cluster 3) N (%)	*p-value*
**Sex**				
Male	9 (22.0)^a^	9 (22.0)^a^	23 (56.1)^b^	0.045
Female	31 (21.2)^a^	61(41.8)^b^	54 (37.0)^b^	
**BMI**				
Low weight	17 (20.0)	28 (32.9)	40 (47.1)	0.663
Eutrophic	13 (23.6)	22 (40.0)	20 (36.4)	
Excess weight	10 (21.3)	20 (42.6)	17 (36.2)	
**Multimorbidities**				
≥ 3 comorbidities	13 (18.8)	28 (40.6)	28 (40.6)	0.729
≤ 2 comorbidities	27 (22.9)	42 (35.6)	49 (41.5)	
**Number of medications**				
≥ 5 medications	21 (23.9)	34 (38.6)	33 (37.5)	0.581
≤ 4 medications	19 (19.2)	36 (36.4)	44 (44.4)	
**Type of LSIE**				
For profit	23 (18.7)^a^	57 (46.3)^b^	43 (35.0)^b^	0.002
Not for profit	17 (26.6)^a^	13 (20.3)^a^	34 (53.1)^b^	
**Age**				
< 80 years	10 (14.1)	26 (36.6)	35 (49.3)	0.095
≥ 80 years	30 (25.9)	44 (37.9)	42 (36.2)	

*BMI* body mass index, *LSIE* long-term institution for the older people.

^a/b^Different letters denote a statistically significant difference between the clusters.

When the ordinal logistic regression of the previously analyzed independent variables was performed, it was possible to observe the net effect of age, LDL, HDL, and triglycerides on inflammation (Table 4). The increase of 1 unit of measurement in LDL, HDL, and triglycerides increased the chances of *inflamm-aging* by 1.5%, 4.1%, and 0.9%, respectively (Cluster 3–1), while long-lived older people (≥ 80 years) had an 84.9% chance of presenting *inflamm-aging* in relation to non-long-lived older people (< 80 years).

**Table 4:** Ordinal logistic regression of independent variables in relation to inflammatory states.

Inflammatory states	Odds ratio	Standard error	Z	p > [z]	CI 95%
Age (≥ 80 years)	1.849	0.57	2.00	0.04	1.01–3.39
LDL (mg/dL)	1.015	0.01	3.66	< 0.01	1.01–1.02
HDL (mg/dL)	1.041	0.02	2.60	0.01	1.01–1.07
Urea (mg/dL)	0.98	0.01	− 1.25	0.21	0.96–1.01
Creatinine (mg/dL)	2.315	1.25	1.56	0.12	0.80–6.67
Triglycerides (mg/dL)	1.009	0.01	2.85	< 0.01	1.00–1.01

*LDL* low density lipoproteins, *HDL* high density lipoproteins.

## Discussion

We initially observed that the multivariate technique of interdependence is a good way to evaluate the inflammatory pattern in older people. This analysis showed three clusters with respect to IL-6, TNF-α, and Zn levels, and the age and concentrations of LDL, HDL, and triglycerides were important to characterize the high inflammatory state group.

The literature presents divergences regarding reference levels of pro-inflammatory cytokines and Zn in different populations^43–45^. In this study we adopted reference values for TNF-α of up to 8.1 pg/mL, for IL-6 < 3.1 pg/mL, and for zinc between 70 and 110 μg/dL. Cluster 1 showed the highest inflammatory state, with concentrations of TNF-α and IL-6 exceeding the reference values and both higher than the mild inflammation state, cluster 2. On the other hand, the values of the mild inflammatory state, cluster 2 were consider "transitory", which is slightly above the reference limit, and only TNF—α levels were higher than the no inflammatory state, cluster 3. In contrast to both high and mild inflammatory state clusters, cluster 3 with no inflammatory state, presented mean and interquartile range values of IL-6 and TNF-α within the reference values.

The process of low-intensity chronic inflammation (*inflamm-aging*) results in a continuous increase in the production of pro-inflammatory cytokines, including IL-6 and TNF-α^3–5^. For example, Xia et al.^42^ proposed that pro-inflammatory cytokines play a key role in the mechanism of *inflamm-aging*, with serum concentrations of IL-6 and TNF-α markers representative of *inflamm-aging*^42^. When we observed the characteristics of cluster 1 in relation to the proinflammatory cytokines, we found higher values of IL-6 (8.15 pg/mL) and TNF-α (12.6 pg/mL) than those observed in another study performed with young and older people, IL-6 (2.57 pg/mL) and TNFα (4.94 pg/mL)^43^. In addition, the IL-6 values found in cluster 1 were lower when compared to severe inflammatory conditions such as sepsis (IL-6 = 64.1 pg/mL) and systemic inflammatory response syndrome (IL-6 = 41.1 pg/mL)^43^, a fact that classifies the high inflammatory state cluster with low intensity inflammation. These results are very important to define the risk group among institutionalized older people, recognizing that patients who do not survive this disease present higher values of IL-6 compared to patients who do survive^30^.

In addition to the increase in the synthesis of proinflammatory cytokines triggered in *inflamm-aging*^1^, the decrease in bioavailability of zinc can be considered an important marker to characterize this condition^46^. Zinc has an important role in reducing the transcription of proinflammatory cytokines, while favoring the transcription of anti-inflammatory cytokines^7,46^. Although all three clusters showed zinc values inside reference levels, the plasma zinc concentrations (76.27 μg/mL) observed in the high inflammatory state cluster were statistically lower than those in the no inflammatory state cluster (98.11 µg/mL). In addition, zinc (F = 116.83) was the variable that best described the clusters, followed by IL-6 (F = 101.09) and TNF-α (F = 72.98), demonstrating the importance of including this anti-inflammatory marker in future studies aimed at identifying *inflamm-aging*. Its reduction, either acute or chronic, can stress an inflammatory state, as shown in studies that point to this variable as an important modulator of inflammatory activity^9,46^.

In contrast to the high inflammatory state cluster, the configuration of the proinflammatory and zinc cytokines in the no inflammatory state cluster presented a framework with a lack of inflammation due to the reduced values of TNF-α and IL-6 and high values of zinc. Mocchegiani et al.^46^ defend the ability of pro-inflammatory cytokines to regulate the bioavailability of zinc observed in the clusters, justifying this antagonistic relationship between these two markers. The cluster with a mild inflammatory state presents characteristics of both clusters, in which the concentration of IL-6 was similar to that presented by the cluster with no inflammatory state and the zinc index was similar to that presented by the cluster with high inflammatory state, whereas the TNF-α value presented an intermediate pattern between the other two clusters.

The deficiency of zinc, common in older people^47^, associated with increased secretion of inflammatory cytokines, also plays an important role in oxidative stress and endothelial cell apoptosis^6^. Cardiovascular diseases are associated with IL-6 and TNF-α, which in turn act in distinct but complementary ways^48^. IL-6 is considered an independent risk factor for the prothrombotic state, whereas TNF-α induces endothelium-dependent relaxation, interfering with the binding and transmigration of leukocytes through the endothelium^48^. The concentrations of IL-6 and TNF-α are associated with dyslipidemia, a factor that compromises endothelial function^5,7^.

Systemic immune system dysregulation may be able to cause acute respiratory distress syndrome (ARDS), with multiple organ failure, and finally leading to death in severe cases of SARS-CoV-2 infection. In addition, inflammatory biomarker dosage is also part of preliminary guidelines for the diagnosis and treatment of coronavirus 2 (SARS-CoV-2) infection^49^. Therefore, there is abundant evidence that certain pro-inflammatory cytokines, such as IL-6 and TNF-α promote poor clinical evaluation and high rates of mortality in patients with COVID-19^30,50^. In addition, zinc deficiency alters key transcription factors and adhesion molecules at LDL receptors, increasing the risk of hypertriglyceridemia, which reinforces the deleterious context for cardiovascular health^6,46^. These findings may explain the greater net effect of LDL and triglycerides on *inflamm-aging* compared to other biochemical variables and reinforce the theory of endothelial injury as a theoretical model to elucidate chronic inflammation with a low intensity characteristic of *inflamm-aging*^51,52^. Regarding HDL, although contradictory, the increase in its plasma concentration was also associated with a high inflammatory state in the ordinal logistic regression model. This is because all groups presented reduced HDL values, with a slightly reduced mean in the no inflammatory state cluster, which was already expected due to their overall reduction in cholesterol and LDL concentrations.

This framework of association with the *inflamm-aging* process is evidenced in the results of the present study, which demonstrated that individuals with a high inflammatory state present higher values of LDL, triglycerides, and total cholesterol, resulting in an increased risk of endothelial injury^48^. This probably occurs considering that small increases in the concentrations of LDL, triglycerides, and total cholesterol are able to promote greater infiltration of lipoproteins in the arterial walls, generating accumulation, and, consequently, endothelial injury^53^. In the mild inflammatory state cluster, the proinflammatory cytokines presented lower values compared to the high inflammatory state cluster, a fact that did not occur in the variables related to the lipid profile. This indicates that even a "transitory" inflammatory state can be related to disruption in the lipid profile, which in turn may induce endothelial injury.

This association of blood fat levels with inflamm-aging becomes even more serious when we consider that the presence of cardiovascular disease is associated with a worse prognosis and mortality^54^. In practical terms, a study by^55^ reported an almost two-fold increase in mortality in patients with cardiovascular disease. With regard to the relationship of inflamm-aging, we can highlight that understanding this inflammatory condition is fundamental for the identification of older people with a higher risk of mortality.

Regarding the sociodemographic variables, we observed that male older people (56.1%) were predominantly in cluster 3, while women were in clusters 2 and 3. The greater presence of women (41.8%) in cluster 2 could be explained by the fact that they present a higher chronic inflammatory state in relation to men, justified by the reduction in the production of estrogen, a hormone with an anti-inflammatory protective effect, caused by menopause, which therefore increases the possibility of low intensity inflammatory conditions^56,57^. In addition, not only a reduction in estrogen, but a high body mass index^58^, associated comorbidities (e.g., cardiovascular disease and diabetes)^59^, and gut microbiota composition^60^ can be factors of an inflammatory condition. With respect to socioeconomic conditions, it is expected that older people residing in not-for-profit LSIEs will present a more debilitated global health state^61^. However, a larger mild inflammatory load was observed in older people from for-profit LSIEs. This type of institution generally groups several socioeconomic classes, which could have resulted in this finding. It is pertinent to seek new strategies for future studies, which use more precise data to analyze this variable, in order to differentiate individuals by using the indicator that best stratifies socioeconomic condition.

Moreover, through the logistic regression analysis, we observed that long-lived older people (≥ 80 years old) had a greater influence on the characterization of *inflamm-aging*. In addition, there is an odds ratio of increased inflammatory states for the older people with high values of LDL, HDL, and triglycerides. This was expected, since this age group presents greater factors of health aggravation. Long-lived older people presented a higher prevalence of arterial hypertension^62^, associated with higher cardiovascular risk and lower functional capacity^63^. In a study conducted with long-lived older Brazilians of both sexes, when analyzing 9 chronic noncommunicable diseases, a prevalence of 67.0% of multimorbidity was observed in long-lived older people compared to those aged less than 80 years^64^. In addition, our study showed that older people with inflamm-aging make up about 25.9% of the sample > 80 years. Therefore, it seems that this is not only caused by the aging process, but that other factors may be associated, such as an increase in adipose tissue. Although we did not find a relationship between BMI and *inflamm-aging* in this study, it is widely understood that adipose tissue increases plasma levels of IL-6 and CRP^65^. Another factor that may be linked is chronic stress exposure to endogenous stress secretion of glucocorticoids, as a proinflammatory^66^.

Furthermore, we can infer that long-lived older people (> 80 years) who present a greater amount of fat in the blood may present a higher relative risk of death. Therefore, it is possible to suggest that mechanisms associated with endothelial injury are associated with inflamm-aging, serving as the basis for future studies on this condition in institutionalized older people and prevention factors to reduce mortality. In this way, public health strategies should direct their efforts to the fight against the control of diseases associated with mechanisms promoting endothelial injuries, considering this as the main factor associated with *inflamm-aging.* A strong point for our study is that the majority of institutionalized older people maintained states between mild and no inflammatory state. This may be justified by the fact that some nursing homes for older people utilize complementary therapies, such as nutraceuticals and lifestyle changes that are suggested as a way to improve health and decrease inflammatory overload^67^. In addition, studies should be performed that seek to establish other diagnostic values for the inflammatory condition of older people, especially those that chronically verify the effect of this condition on the health problems of older people. It is important to highlight one study limitation. We only measured zinc as an anti-inflammatory biomarker, although there are other good anti-inflammatory biomarkers, such as interleukin 10 (Il-10)^68^. However, zinc dosage is cheap and is part of some routine exams which is why it was chosen to be used in this study. Therefore, we suggest the analysis of biochemical markers and anti-inflammatory markers as a way to monitor other health-related factors and decrease future costs of a preventive evaluation for groups at higher risk.

## Conclusion

IL-6, TNFα, and zinc were shown to be responsive markers for characterizing *inflamm-aging* in institutionalized older people. Individuals with a high inflammatory state present higher concentrations or altered lipid profiles of HDL, LDL, and triglycerides in relation to individuals with a reduced inflammatory state, demonstrating the association of the lipid profile with a high inflammatory state. With regard to sociodemographic aspects, it was observed that long-lived older people showed the greatest influence from *inflamm-aging.*
